# PLK1 Inhibitor Onvansertib Enhances the Efficacy of Alpelisib in *PIK3CA*-Mutated HR-Positive Breast Cancer Resistant to Palbociclib and Endocrine Therapy: Preclinical Insights

**DOI:** 10.3390/cancers16193259

**Published:** 2024-09-25

**Authors:** Sreeja Sreekumar, Elodie Montaudon, Davis Klein, Migdalia E. Gonzalez, Pierre Painsec, Héloise Derrien, Laura Sourd, Tod Smeal, Elisabetta Marangoni, Maya Ridinger

**Affiliations:** 1 Cardiff Oncology Incorporated, San Diego, CA 92121, USA; ssreekumar@cardiffoncology.com (S.S.); dklein@cardiffoncology.com (D.K.); dgonzalez@cardiffoncology.com (M.E.G.); tsmeal@cardiffoncology.com (T.S.); 2 Laboratory of Preclinical Investigation, Translational Research Department, Institut Curie, 75005 Paris, France; elodie.montaudon@curie.fr (E.M.); pierrepainsec@gmail.com (P.P.); heloise.derrien@curie.fr (H.D.); laura.sourd@curie.fr (L.S.); elisabetta.marangoni@curie.fr (E.M.)

**Keywords:** breast cancer, cyclin-dependent kinase inhibitor, endocrine therapy, hormone receptor, metastatic breast cancer, onvansertib, patient-derived xenografts, PLK1

## Abstract

**Simple Summary:**

Patients with hormone receptor-positive (HR+)/HER2-negative metastatic breast cancer who have *PIK3CA* mutations and have progressed on first-line therapies are considered for treatment with alpelisib alongside endocrine therapy (ET). Combination therapeutic strategies may extend the clinical benefit of alpelisib. This study investigated the potential of onvansertib, a highly specific inhibitor of polo-like kinase 1 (PLK1), to enhance the efficacy of alpelisib in preclinical models of HR+ breast cancer. Our findings demonstrated that onvansertib synergizes with alpelisib in *PIK3CA*-mutant HR+ breast cancer cell lines and patient-derived xenografts that are resistant to ET and cyclin-dependent kinase 4/6 inhibitors. The combination inhibited PLK1 and PI3K signaling, induced cell cycle arrest, and triggered apoptotic death in both cell lines and xenograft tumors. Overall, our preclinical data encourage the clinical exploration of this combination for *PIK3CA*-mutant HR+ metastatic breast cancer patients progressing on standard-of-care therapies.

**Abstract:**

Background: Endocrine therapy (ET) combined with cyclin-dependent kinase 4/6 inhibitors (CDK4/6i) is the preferred first-line treatment for hormone receptor-positive (HR+)/HER2- metastatic breast cancer. Although this is beneficial, acquired resistance leads to disease progression, and patients harboring *PIK3CA* mutations are treated with targeted therapies such as the PI3Kα inhibitor, alpelisib, alongside ET. Drug-associated resistance mechanisms limit the efficacy of alpelisib, highlighting the need for better combination therapies. This study aimed to evaluate the efficacy of combining alpelisib with a highly specific PLK1 inhibitor, onvansertib, in *PIK3CA*-mutant HR+ breast cancer preclinical models. Methods: We assessed the effect of the alpelisib and onvansertib combination on cell viability, PI3K signaling pathway, cell cycle phase distribution and apoptosis in PI3K-activated HR+ breast cancer cell lines. The antitumor activity of the combination was evaluated in three *PIK3CA*-mutant HR+ breast cancer patient-derived xenograft (PDX) models, resistant to ET and CDK4/6 inhibitor palbociclib. Pharmacodynamics studies were performed using immunohistochemistry and Simple Western analyses in tumor tissues. Results: The combination synergistically inhibited cell viability, suppressed PI3K signaling, induced G2/M arrest and apoptosis in PI3K-activated cell lines. In the three PDX models, the combination demonstrated superior anti-tumor activity compared to the single agents. Pharmacodynamic studies confirmed the inhibition of both PLK1 and PI3K activity and pronounced apoptosis in the combination-treated tumors. Conclusions: Our findings support that targeting PLK1 and PI3Kα with onvansertib and alpelisib, respectively, may be a promising strategy for patients with *PIK3CA*-mutant HR+ breast cancer failing ET + CDK4/6i therapies and warrant clinical evaluation.

## 1. Introduction

Breast cancer is the most prevalent cancer in women and a leading cause of cancer-related deaths in women worldwide [1]. Approximately 70% of breast cancers are hormone receptor-positive (HR+). The standard-of-care for HR+ metastatic breast cancer typically involves treatment with endocrine therapy (ET) in combination with cyclin-dependent kinase 4/6 inhibitors (CDK4/6i) such as palbociclib, ribociclib, or abemaciclib. However, most patients eventually progress due to acquired resistance to ET and/or CDK4/6i [2]. Following progression, alternative therapies are actively being explored, including antibody–drug conjugates, immunotherapies, kinase inhibitors, and novel anti-estrogens to improve outcomes [3,4]. Eligible patients are treated with inhibitors targeting the PI3K/AKT/mTOR pathway, such as alpelisib, capivasertib, and everolimus, together with ET [4]. However, de novo and acquired resistance to these inhibitors, as well as off-target toxicities, pose significant clinical challenges [4,5,6,7]. The PI3K/AKT/mTOR pathway is a critical regulator of cell proliferation and survival [8,9]. The pathway is often altered in HR+ breast cancer, and its constitutive activation contributes to disease progression and resistance, making the pathway an attractive therapeutic target [10]. Activating mutations in *PIK3CA*, the gene encoding the p110α catalytic domain of PI3K, is one of the frequently altered genes in HR+ breast cancer (~40%) and is implicated in resistance to therapy [11,12,13]. PI3Kα inhibitor, alpelisib, in combination with the ET fulvestrant, is approved for *PIK3CA*-mutated, advanced HR+ breast cancer [14]. However, drug-associated toxicities and resistance mechanisms, including loss of PTEN function, may hamper its clinical benefit [15,16,17]. Thus, there is a need to explore additional molecular targets and combinatorial treatments to increase efficacy and overcome therapy resistance.

The serine/threonine protein kinase polo-like kinase 1 (PLK1) is found to be overexpressed in breast cancer and has been associated with poor prognosis [18,19]. PLK1 plays a key role in cell cycle progression, G2/M phase transition, mitotic progression, cytokinesis, and the DNA damage response [20,21,22]. Notably, PLK1 was found to be upregulated in bone metastasis-derived patient-derived xenograft (PDX) models from HR+ breast cancer patients who progressed on ET compared to their matched primary tumors [23]. The pan-PLK inhibitor volasertib showed profound anti-tumor activity in these CCND1-driven PDX models, with acquired resistance to palbociclib, including those with *PIK3CA* mutations [23]. Moreover, a recent study reported that elevated levels of *PLK1* mRNA were associated with poorer response to palbociclib plus ET in HR+ breast cancer patients [24]. Interestingly, PLK1 has been reported to regulate the PI3K/AKT/mTOR pathway in multiple cancers [25,26,27,28,29]. However, there are no comprehensive studies investigating the crosstalk between PLK1 and PI3K pathways in breast cancer. Elevated levels of PLK1 have been shown to activate the PI3K/AKT/mTOR pathway, leading to castration-resistant prostate cancer (CRPC) [29]. Furthermore, combining PI3K inhibition with PLK1 depletion has been reported to enhance chemo-sensitization in pancreatic cancer cells [26]. Collectively, these studies underscore the therapeutic promise of combined targeting of PI3K and PLK1 pathways for combating therapy resistance and improving the clinical outcome of patients with advanced HR+ breast cancer.

Onvansertib is an orally bioavailable, highly selective ATP-competitive PLK1 inhibitor currently in clinical development [30]. Onvansertib is currently being evaluated as a first-line therapy for metastatic colorectal cancer (mCRC) in a phase 2 trial (NCT06106308). A phase 1b/2 clinical trial (NCT05383196) is ongoing to evaluate the safety and efficacy of onvansertib and paclitaxel combination in advanced triple-negative breast cancer (TNBC). Preclinically, onvansertib has been demonstrated to be effective both as a single agent and in combination with either chemotherapeutics or targeted therapies in ovarian cancer, prostate cancer, triple-negative breast cancer (TNBC), and hematological malignancies [31,32,33,34]. Though onvansertib was found to be effective in HR+ breast cancer cell lines [35], to the best of our knowledge, no study has evaluated its activity *in vivo* in HR+ breast cancer models.

We investigated the combination of alpelisib and onvansertib in *PIK3CA*-mutant HR+ metastatic breast cancer models. We evaluated the synergistic efficacy of the combination in ET-sensitive and -resistant cell lines and PDX models that are resistant to palbociclib and ET. The combination displayed robust anti-tumor activity compared to the monotherapies in all the preclinical models tested. Mechanistic studies revealed pronounced G2/M arrest and apoptosis with the combination, both *in vitro* and *in vivo*. Our findings suggest that the novel combination of alpelisib and onvansertib presents a promising therapeutic strategy and warrants clinical exploration of this regimen for HR+ breast cancer patients progressing on CDK4/6i and ET.

## 2. Materials and Methods

### 2.1. Cell Lines and Culture Conditions

Human estrogen receptor-positive (ER+)/HER2- breast cancer cell lines CAMA-1, HCC1428, MCF7, T-47D, and ZR-75-1 were obtained from American Type Culture Collection (ATCC, Manassas, VA, USA). The EFM-19 cell line was purchased from Leibniz Institute DSMZ (Braunschweig, Germany). All the cell lines except MCF7 were cultured in RPMI-1640 (30-2001; Thermo Fisher, Waltham, MA, USA), supplemented with 10% fetal bovine serum (FBS; #16000044; Thermo Fisher). MCF7 was grown in high-glucose Dulbecco’s modified Eagle medium (DMEM; 11965092; Thermo Fisher) supplemented with 10% FBS. MCF7/164R-7, fulvestrant-resistant cell line, was purchased under license from CancerTools and was cultured in phenol red-free DMEM (11039021; Thermo Fisher) supplemented with 1% FBS and 100 nM fulvestrant. The drug was removed 24 h prior to the experiments. The cell lines were maintained at 37 °C, 5% CO_2_ in a humidified incubator. The cell lines were confirmed negative for Mycoplasma using the MycoAlert Mycoplasma Detection kit (LT07-318; Lonza, Rockland, ME, USA).

### 2.2. Cell Viability and Synergy Analysis

Breast cancer cells were seeded at a density of 400–750 cells/well in 384-well plates, and on the next day, cells were treated with varying doses of onvansertib (10^−11.5^ to 10^−6^ M) or alpelisib (10^−11.5^ to 10^−5^ M). DMSO-treated cells served as control. The DMSO concentration was maintained below 0.01% and was kept constant in all the wells. After incubating for 6–7 days, cell viability was determined using the CellTiter Glo Cell Viability kit (G9243; Promega, Madison, WI, USA) following the manufacturer’s instructions. Luminescence was measured using an Infinite Lumi plate reader (Tecan, Männedorf, Switzerland). The luminescence of control cells was considered 100%. The half-maximal inhibitory concentration (IC50) values were determined using GraphPad Prism 10.0 software (GraphPad Software, Boston, MA, USA). Synergy studies were performed using 8 concentrations of each drug and combinations between them in a dose–response matrix. Synergistic efficacy was assessed using the Combenefit software version 2.021 based on the Bliss independence drug combination model [36].

### 2.3. Colony Formation Assay

The cells were plated in 12-well plates at a density of 800–1000 cells/well in triplicates, allowed to attach, and treated with vehicle (0.01% DMSO) or the indicated drugs. After incubating for 72 h to 144 h, the cells were washed twice with growth medium and cultured for 10–22 days, with drug-free medium added every three days. The colonies formed were fixed and stained with crystal violet, as reported previously [37]. The colony formation was quantified using the ColonyArea ImageJ plugin (version 1.53t, NIH) as per the published protocol [38].

### 2.4. Flow Cytometric Analysis of Cell Cycle Distribution and Apoptosis

The effect of onvansertib, alpelisib, or their combination in altering cell cycle phase distribution and apoptosis was analyzed by flow cytometry. After treating the cells with the drugs for the indicated durations, cells were harvested, fixed in BD Cytofix™ Fixation Buffer (554655; BD Biosciences, San Jose, CA, USA), and stained with 4′,6-diamidino-2-phenylindole (DAPI 1 μg/mL, Triton X100 0.1%) for DNA content analysis. The effect of the drugs in inducing apoptotic DNA fragmentation was evaluated using APO-DIRECT™ Apoptosis Detection Kit (556381; BD Biosciences) according to the manufacturer’s instructions. The method utilizes a reaction catalyzed by exogenous terminal deoxynucleotidyl transferase (TdT) for labeling DNA breaks with fluorescein isothiocyanate-labeled dUTP (FITC-dUTP), followed by flow cytometric analysis. The process is referred to as TdT dUTP nick end labeling (TUNEL) [39]. Briefly, cells were harvested after indicated treatments, washed, and fixed in 1% (*w*/*v*) paraformaldehyde in PBS (pH 7.4). After fixing the cells in 70% (*v/v*) ice-cold ethanol at −20 °C for at least 18 h, the DNA breaks were labeled with FITC-dUTP using the TdT enzyme. The percentage of FITC-dUTP-positive cells or DAPI intensity defining cell cycle phase distribution (G0/G1, S, and G2/M) was measured by flow cytometry using FACSCelesta™ Cell Analyzer (BD Biosciences) equipped with the BD FACSDiva™ Software (version 9; BD Biosciences). The acquired data were analyzed using FlowJo™ Software version 10.9 (BD Biosciences).

### 2.5. Protein Analysis Using Simple Western

After the indicated treatments, cell or tumor protein lysates were prepared in lysis buffer (10 mM Tris-HCl (pH 7.4), 25 mM β-glycerophosphate, 150 mM NaCl, 20 mM NaF, 1 mM Na3VO4, 5 mM EDTA, 1 mM Na4P2O7, 10% glycerol, 1% IGEPAL CA-630) containing 1× protease (P8340; Sigma Aldrich, St. Louis, MO, USA), and phosphatase inhibitors (P5726; Sigma Aldrich). Total protein concentration was determined using the Pierce BCA Protein Assay Kit (23227; Thermo Fisher). The lysates were analyzed using the Protein Simple Jess size-based capillary electrophoresis system (Bio-Techne, Minneapolis, MN, USA) according to the manufacturer’s instructions. Briefly, the protein lysates were diluted with sample buffer to a concentration of 1 mg/mL and then mixed with 5× fluorescent master mix at a 4:1 ratio and heated at 95 °C for 5 min. The samples (3 µL each) were then loaded into a 12–230 kDa separation cassette. The size-separated proteins were probed with primary antibodies in antibody buffer followed by HRP-conjugated secondary antibodies (Table S1). Data were analyzed using Compass for Simple Western or ImageJ.

### 2.6. In Vivo PDX Studies

Palbociclib-resistant *PIK3CA*-mutant HR+ breast cancer PDXs were established from primary breast tumor (HBCx-86) or from metastatic bone biopsies of patients who had progressed on ET plus palbociclib (HBCx-180) or PI3Kα inhibitor alpelisib (HBCx-134palboR31) as described previously [23,40,41,42]. All animal experimental procedures were approved by the Ethics Committee on Animal Experimentation of the Institute Curie (National registration no: #118, authorization no: 02163.02). Animal care and the experimental procedures were conducted in strict accordance with the recommendations of the European Community Directive (2010/63/UE) for the care and use of laboratory animals. Female Swiss nude mice, 6 to 8 weeks old, from Charles River Laboratories maintained at Institut Curie in pathogen-free conditions were utilized for the studies. PDX tumors were grafted into the interscapular fat pad of mice as previously described [43]. The mice were randomized to different treatment groups (*n* = 6–8 per group) once the tumors reached a volume between 60 and 200 mm^3^. The animals were treated with vehicle, onvansertib (45 mg/kg, oral, 5 days a week), alpelisib (25 mg/kg, oral, 5 days a week), or the combination for 32 days. Alpelisib (HY-15244; MedchemTronica, Stockholm, Sweden) and onvansertib (Cardiff Oncology, San Diego, CA, USA) were suspended in 0.5% methylcellulose containing 0.1% Tween80. Tumor sizes were measured twice per week using a caliper. The tumor volumes were calculated as *V* (mm^3^) =  *a*  ×  *b*^2^/2, a being the largest diameter, *b* the smallest and perpendicular to *a*. Relative tumor volume (RTV) was calculated as RTV = (tumor volume on measured day)/(tumor volume on day 0). An RTV of <0.5 was classified as regression, 0.5 to 1.35 as stable disease, and >1.35 as progressive disease [23].

### 2.7. Pharmacodynamic Studies

HBCx-86 PDX tumors were harvested from the animals at the end of the efficacy study and fixed in 10% neutral buffered formalin (NBF) for histopathological analysis. HBCx-134palboR31 xenograft-bearing mice were randomly allocated into treatment groups (*n* = 3–4 per group) once the mean tumor volume reached 63 to 172 mm^3^. The animals were treated with vehicle, onvansertib (45 mg/kg), alpelisib (25 mg/kg), or the combination. The first day of treatment was designated as day 0. Tumor samples were collected on day 4, 3 h post-treatment, and one-half of the tissues were flash frozen and stored at −80 °C for protein extraction and analysis, and the other halves were fixed in 10% NBF for histology analysis. The samples were dehydrated, embedded in paraffin, and sectioned at 4 μm. The deparaffinized tissue sections were stained with hematoxylin and eosin (H&E) at HistoWiz (Brooklyn, NY, USA) using in-house automated standard operating procedures. Whole-slide scanning was performed using an Aperio AT2 imaging system (Leica Biosystems, Vista, CA, USA). The digital images were examined, and apoptosis was evaluated by a senior board-certified pathologist. Apoptotic cells were manually counted in five randomly selected fields per tumor section, excluding necrotic regions and surrounding stroma, observed at 40× magnification.

### 2.8. Statistical Analysis

Statistical analyses were performed using Graph Pad Prism 10 software. Data are presented as mean ± standard error of mean (SEM), calculated from at least three independent biological replicate experiments or sample numbers. Comparisons between groups were made using an unpaired *t*-test (two groups) or one-way/two-way ANOVA (three or more groups) with Tukey’s multiple comparison test. *In vivo* efficacy studies were evaluated using Kaplan–Meier survival curves compared with the log-rank (Mantel–Cox) test. *p* values < 0.05 were considered significant and are indicated by asterisks in figures (****, *p* < 0.0001; ***, *p* < 0.001; **, *p* < 0.01; *, *p* < 0.05).

## 3. Results

### 3.1. Onvansertib and Alpelisib Are Synergistic in ER+ Breast Cancer Cell Lines

**Figure 1:**
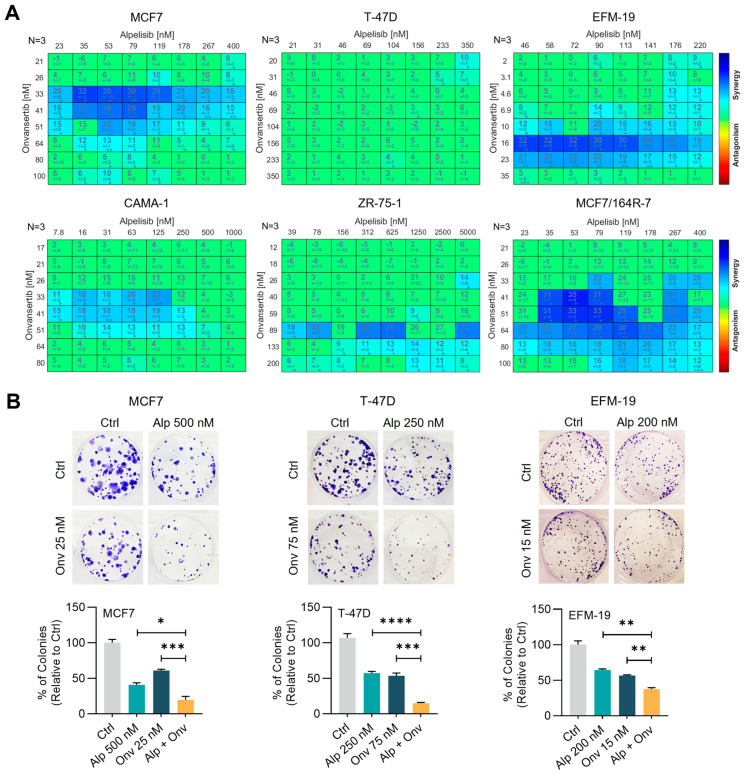
Combination of alpelisib and onvansertib displays a synergistic effect in HR+ breast cancer cell lines. (**A**) Cell viability was assessed after 6–7 days of treatment with onvansertib (Onv), alpelisib (Alp), or the combination of Onv + Alp at the indicated concentrations, and synergy was assessed. Heatmaps of the combination responses for onvansertib and alpelisib based on the Bliss synergy model are shown. Blue color indicates synergistic interaction (*n* = 3). (**B**) Colony formation assay of MCF7, T-47D, and EFM-19 cells treated with Alp and Onv or their combination (Alp + Onv) at the indicated concentrations. Representative wells are shown in the top panel, and percentages of colonies normalized to the DMSO-treated control (Ctrl) from three independent experiments are shown in the bottom panel. Results presented as mean ± SEM. Asterisks indicate significance by one-way ANOVA (* *p* < 0.05, ** *p* < 0.01, *** *p* < 0.001, **** *p* < 0.0001).

We selected three *PIK3CA*-mutant (MCF7, EFM-19, and T-47D) and two *PIK3CA*-wildtype *PTEN*-lost (ZR-75-1 and CAMA-1) ER+ breast cancer cell lines for the study. The fulvestrant-resistant derivative of MCF7 cell line MCF7/164R-7 was also included. First, we evaluated the effect of onvansertib and alpelisib single agents on the viability of all six cell lines and determined the half-maximal inhibitory concentration (IC_50_) values (Table S2). Irrespective of the mutational status, onvansertib dose-dependently decreased the viability of all the tested cell lines with IC_50_ values ranging from 23 to 211 nM. Consistent with previous studies [44], alpelisib decreased the viability of *PIK3CA*-mutant cell lines (IC50 ranging from 185 to 288 nM), while the *PIK3CA*-wildtype cell lines with *PTEN* loss displayed significantly less sensitivity (IC50 > 1 µM) to the drug. We then assessed the effect of combining onvansertib and alpelisib on cell viability in a 9 × 9 dose–response matrix using the Bliss independence synergy model. In five cell lines (MCF7, EFM-19, ZR-75-1, CAMA-1, and MCF7/164R-7), the combination of onvansertib and alpelisib was synergistic, as shown by high Bliss synergy scores, while the combination showed no synergy in the sixth cell line (T-47D) (Figure 1A and Figure S1). We also tested the effect of onvansertib in combination with inavolisib, a selective inhibitor and degrader of mutant PI3Kα that is currently under clinical evaluation for *PIK3CA*-mutant breast cancer [45]. In both the tested cell lines, MCF7 and EFM-19, the combination of onvansertib and inavolisib was found to be synergistic in decreasing the cell viability (Figure S2).

Earlier reports suggested that alpelisib decreases the colony-forming ability of breast cancer cell lines [46,47]. We examined the effect of alpelisib and onvansertib on the colony-forming ability of MCF7, T-47D, and EFM-19 *PIK3CA*-mutant cell lines (Figure 1B). All three cell lines exhibited significantly decreased clonogenicity upon treatment with the onvansertib and alpelisib combination compared to single agents, attesting to the long-term beneficial effects of the combination. The effect was also observed in T-47D cells (Figure 1B) despite the lack of synergistic response in the cell viability assay (Figure 1A). Collectively, these data indicate that combined inhibition of PLK1 and PI3K synergistically inhibits cell proliferation and impairs the clonogenicity of ER+ breast cancer cell lines harboring PI3K-pathway alterations.

### 3.2. The Combination of Onvansertib and Alpelisib Suppresses PI3K-AKT Signaling, Induces G2/M Arrest, and Apoptosis

We then evaluated the effect of the onvansertib and alpelisib combination on the PI3K/AKT/mTOR signaling pathway by assessing the protein expression of downstream targets of PI3K (pAKT-Ser473, pGSK3β-Ser9, and pS6-Ser240/244) in MCF7 cells. After treating the cells for 4 h, alpelisib single agent and its combination with onvansertib effectively inhibited the PI3K pathway, as shown by the significant decrease in pAKT-Ser473, pGSK3β-Ser9, and pS6-Ser240/244 levels (Figure S3). There was a greater reduction in pAKT-Ser473 and pS6-Ser240/244 levels upon 24 h of treatment with the combination compared to the individual drugs, although the difference with the alpelisib treatment was not statistically significant (Figure S3). These results demonstrate that the combination treatment induced sustained inhibition of the PI3K/AKT/mTOR signaling pathway.

**Figure 2:**
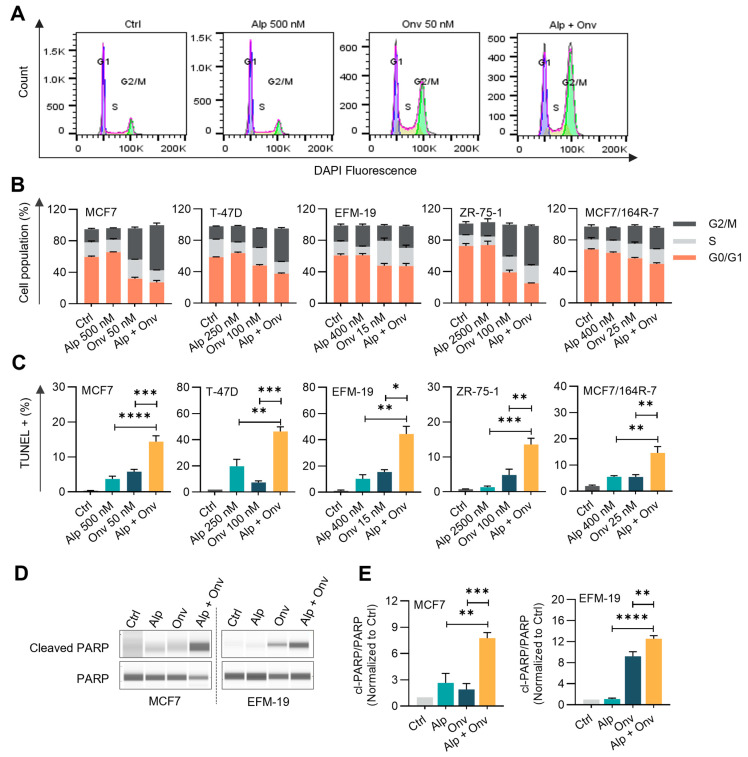
Alpelisib and onvansertib combination induces G2/M arrest and apoptosis in ER+ breast cancer cell lines. (**A**,**B**) The cells were treated with alpelisib (Alp), onvansertib (Onv), or the combination (Alp + Onv) for 72–96 h at the indicated concentrations, and cell cycle analysis was performed. (**A**) Representative DNA histograms showing the population of cells in the G1, S, and G2/M cell cycle phases of MCF7 treated with the drugs. (**B**) The bar graph showing percentage of cells in the G1, S, and G2/M phases of MCF7, T-47D, EFM-19, ZR-75-1, and MCF7/164R-7 cells after treating with Onv, Alp or Alp + Onv. (**C**) The cells were treated with Onv, Alp, or the combination at the indicated concentrations, and the percentage of apoptotic cells was analyzed by a TUNEL assay. The percentage of TUNEL+ cells is plotted. (**D**) MCF7 and EFM-19 cells were treated with DMSO vehicle (Ctrl), Onv (25 nM for MCF7, 15 nM for EFM-19), Alp (200 nM for MCF7, 100 nM for EFM-19), or Onv + Alp for 24 h. Representative Simple Western images of cleaved-PARP (cl-PARP) and total PARP protein expression. The uncropped Simple Western images are shown in Figure S6. (**E**) Densitometric ratio of cleaved-PARP to total PARP expression levels normalized to DMSO control are plotted. All results are the mean of three experiments and are presented as mean ± SEM. One-way ANOVA was used to compare the means. Asterisks indicate significance (* *p* < 0.05, ** *p* < 0.01, *** *p* < 0.001, **** *p* < 0.0001).

We examined the effect of onvansertib and alpelisib on the cell cycle phase distribution. Cell cycle analysis was performed by flow cytometry analysis on four *PIK3CA*-mutant (MCF7, EFM-19, T-47D, and MCF7/164R-7) and one *PIK3CA*-wildtype, *PTEN*-lost (ZR-75-1) cell lines (Figure 2A,B). Alpelisib, at the tested concentrations, did not alter the cell cycle distribution of the tested cell lines (Figure 2B). As expected, onvansertib treatment induced G2/M arrest in four out of five cell lines (Figure 2B). The exception was the EFM-19 cell line in which onvansertib treatment resulted in an increase in cells in the S-phase (Figure 2B and Figure S4A). Similarly, siRNA-mediated inhibition of PLK1 arrested EFM-19 cells in the S-phase (Figure S4B,C). In all the other tested cell lines, the combination of onvansertib and alpelisib induced more pronounced G2/M arrest compared to onvansertib monotherapy (Figure 2B).

To determine whether G2/M arrest induced by the combination led to cell death, we assessed the effect of the combination on apoptosis. In all the cell lines, including EFM-19, the combination resulted in a marked increase in apoptosis (TUNEL+ cells) compared to the control and single agents (Figure 2C). Moreover, an increase in apoptotic marker cleaved-PARP-1 was observed in MCF7 and EFM-19 cells treated with the combination compared to either onvansertib or alpelisib single agents and DMSO control (Figure 2D,E). Overall, the findings indicate that the combination of onvansertib and alpelisib induced cell cycle perturbations, resulting in increased apoptosis in HR+ breast cancer cells.

### 3.3. Co-Administration of Onvansertib and Alpelisib Inhibits the Growth of PIK3CA-Mutated, ET, and Palbociclib-Resistant HR+ Breast Cancer PDXs

We then assessed the combination of onvansertib and alpelisib in three HR+ (ER+) breast cancer PDX models. For this purpose, palbociclib-resistant *PIK3CA*-mutant breast cancer PDXs were established from primary breast tumor (HBCx-86; *PIK3CA*-E545K) or from metastatic bone biopsies of patients who had progressed on ET combined with palbociclib (HBCx-180; *PIK3CA*-H1047R) or alpelisib (HBCx-134palboR31; *PIK3CA*-H1047R) [23,41]. HBCx-86 and HBCx-180 PDXs displayed intrinsic resistance to palbociclib, while HBCx-134palboR31 PDX is an acquired resistance model established by long-term exposure to palbociclib [23]. HBCx-180 and HBCx-134palboR31 PDXs were resistant to the combination of palbociclib and fulvestrant, while HBCx-86 PDX showed stable disease with the treatment [23].

**Figure 3:**
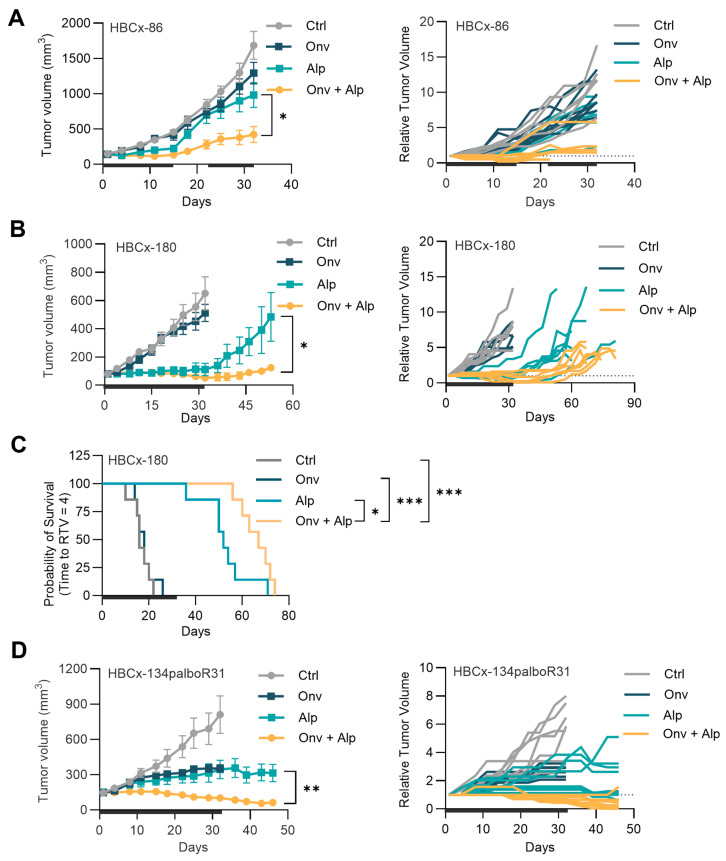
The combination of onvansertib and alpelisib shows robust anti-tumor activity *in vivo*. (**A**) HBCx-86, (**B**,**C**) HBCx-180, and (**D**) HBCx-134palboR31 PDX models were treated with vehicle (Ctrl), onvansertib (Onv, 45 mg/kg), alpelisib (Alp, 25 mg/kg), or combination of Alp and Onv (*n* = 6 to 8 per group) for the indicated duration (—). (**A**,**B**,**D**) Mean ± SEM tumor volumes are shown on the left, and individual relative tumor volumes (RTV) are shown on the right. Tumor regression is reported if RTV < 0.5 in at least 1 tumor measurement. An unpaired *t*-test was used to compare RTV at the last measurement. (**C**) Kaplan–Meier survival curve for event-free survival (time for RTV = 4) was calculated. Log-rank Mantel–Cox test was used for survival analyses * *p* < 0.05, ** *p* < 0.01, *** *p* < 0.001.

In the HBCx-86 PDX, although neither onvansertib nor alpelisib showed a significant reduction in tumor volume compared to the vehicle-treated control, the combination induced potent tumor growth inhibition and stabilized the tumors in 40% of mice (4/10) (Figure 3A). Next, we assessed the effect of the combination in HBCx-180, a PDX established from a patient with intrinsic resistance to palbociclib. We observed that in the HBCx-180 PDX, the combination treatment induced a profound reduction in tumor volume with tumor regression in 57% of mice (4/7), while only 14% (1/7) of mice treated with alpelisib displayed tumor regression, and the tumors were resistant to onvansertib single agent (Figure 3B).The efficacy of the combination was greater and more durable compared to the alpelisib single agent, with significant event-free survival (Log-rank test, *p* < 0.05) (Figure 3C). Finally, we tested the combination in the PDX HBCx-134palboR31, established from bone metastasis of a breast cancer patient treated with alpelisib in the neo-adjuvant setting and who failed to respond to it. The PDX model showed resistance to alpelisib when combined with fulvestrant [23]. Notably, in this PDX, the combination treatment induced pronounced tumor regression in 75% of mice (6/8), with complete response in 25% (2/8) of mice, while only 1 out of the 8 alpelisib treated mice and none of the onvansertib-treated mice showed tumor regression (Figure 3D).

The combination was well tolerated, with mean body weight loss of less than 10% in HBCx-86 and HBCx-134palboR31 models (Figure S5). In the HBCx-180 model, in the combination-treated group, mean body weight loss of >10% was observed, but the animals recovered with cessation of treatment (Figure S5). Overall, the combination of onvansertib and alpelisib was well tolerated and showed significant anti-tumor activity, which was superior to the single agents in all three PDX models.

### 3.4. Onvansertib and Alpelisib Combination Inhibits PLK1 and PI3K Activity and Induces Pronounced Apoptosis in the Combination-Treated Tumors

**Figure 4:**
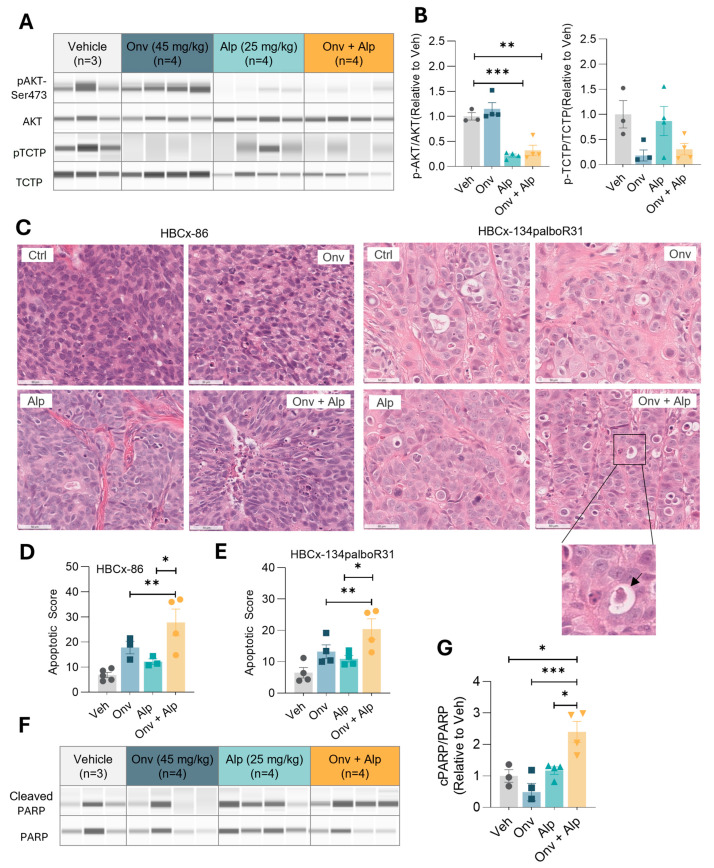
Onvansertib and alpelisib combination inhibits the PLK1 and PI3K pathways and induces apoptosis *in vivo*. (**A**) Protein levels of pAKT-Ser473, AKT, phospho-TCTP, and TCTP in HBCx-134palboR31 tumors treated with vehicle (Veh), onvansertib (Onv), alpelisib (Alp), or the combination (Onv + Alp) for 4 days. (**B**) The normalized densitometric ratio of the phospho-proteins to total protein levels is plotted. (**C**) H&E-stained photomicrographs (40×) showing apoptotic cells in HBCx-86 and HBCx-134palboR31 tumors treated with Veh, Onv, Alp, or Alp + Onv for 32 days (HBCx-86) or 4 days (HBCx-134palboR31). (**D**,**E**) Apoptotic cells were manually counted and plotted. (**F**) Cleaved-PARP and total PARP protein expression in HBCx-134palboR31 tumors. (**G**) Graphs show normalized densitometric ratio of cleaved-PARP to total PARP. The uncropped Simple Western images are shown in Figure S6. Data are expressed as mean ± SEM. One-way ANOVA was used to compare the means. Asterisks indicate significance (* *p* < 0.05, ** *p* < 0.01, *** *p* < 0.001).

To evaluate *in vivo* target inhibition of PLK1 and PI3Kα kinases, we assessed the protein and phospho-protein expression levels of TCTP (pTCTP), a direct substrate of PLK1, and AKT (pAKT-Ser473), an index of PIK3 activation in HBCx-134palboR31 tumors. As expected, the relative levels of pTCTP were downregulated in tumors treated with onvansertib, and pAKT was decreased in tumors treated with alpelisib (Figure 4A,B). In tumors treated with the combination, both pTCTP and pAKT were decreased, confirming the inhibition of the PLK1 and PI3K pathways (Figure 4A,B).

As our *in vitro* studies showed an increased apoptosis in cells treated with the combination, we examined apoptotic markers in tumors. Compared to the alpelisib and onvansertib monotherapies, the combination induced increased apoptosis in HBCx-86 and HBCx-134palboR31, as evidenced by cell shrinkage and nuclear condensation (Figure 4C–E). The apoptosis was further confirmed in the HBCx-134palboR31 PDX model by Western blot analysis of cleaved-PARP (Figure 4F,G). The cleaved-PARP protein levels were found to be increased in the combination group (Onv + Alp) compared to the monotherapies, attesting the induction of apoptosis in these tumors resistant to alpelisib plus fulvestrant.

## 4. Discussion

Although there are multiple targeted therapies for HR+ metastatic breast cancer, new agents and combination therapies are needed to address the eventual development of resistance to current therapies [46,48]. Efforts are also focused on improving the early-stage diagnosis of HR+ breast cancer to enhance treatment outcomes and overall prognosis [49,50,51]. In this study, we have investigated the therapeutic potential of the PLK1 inhibitor onvansertib in combination with the FDA-approved PI3Kα inhibitor alpelisib for advanced HR+ breast cancer. We demonstrated that the combination synergistically decreases the survival of HR+ breast cancer cells harboring *PIK3CA* mutations or *PTEN* loss and elicits robust anti-tumor activity in *PIK3CA*-mutant PDX models resistant to alpelisib and/or palbociclib combined with endocrine therapy. This study is the first to evaluate the efficacy of onvansertib, both as a monotherapy and in combination with PI3K inhibitors, in *in vivo* models of HR+ breast cancer. To the best of our knowledge, this is also the first report of the synergistic effect between inhibitors targeting the PI3K and PLK1 pathways in HR+ breast cancer.

Previous investigations provided a compelling rationale for targeting PLK1 in HR+ breast cancer. PLK1 expression is reported to be associated with worse prognosis and resistance to CDK4/6i in patients with HR+ metastatic breast cancer [24]. The pan-PLK inhibitor volasertib demonstrated efficacy as a single agent in HR+ breast cancer PDX models resistant to CDK4/6i [23]. However, in our study, onvansertib as a single agent only showed anti-tumor activity in one of the three models tested, highlighting the need to explore combination therapies. In the clinical setting, PLK1 inhibitors have shown limited activity as monotherapy thus far, and current clinical investigations focus on combinations with targeted therapies and chemotherapeutics [22]. In a recent study, onvansertib, when combined with the standard-of-care FOLFIRI and bevacizumab, was found to be well tolerated and showed a promising signal of efficacy in *KRAS*-mutated metastatic colorectal cancer patients [52], supporting the strategy of combining PLK1 inhibitors with approved therapies to enhance their effectiveness.

Resistance to CDK4/6i and ET for HR+ breast cancer is found to be associated with alterations in the PI3K/AKT/mTOR pathway [53]. Alpelisib plus fulvestrant displayed an overall response rate (ORR) of 19.0% and median progression-free survival (PFS) around 11 months in *PIK3CA*-mutated HR+ advanced breast cancer patients progressing on or after treatment with CDK4/6i and/or ET [6,54]. Exploring the combination of alpelisib with onvansertib stemmed from the literature indicating crosstalk between PLK1 and PI3K pathways, suggesting that inhibiting both pathways can combat resistance to therapy [25,26,27,29]. We have observed a strong synergistic effect when onvansertib is combined with alpelisib in HR+ cell lines carrying *PIK3CA* mutations or *PTEN* loss. The fulvestrant-resistant model also exhibited heightened sensitivity to the combination compared to the single agents. Onvansertib enhanced the efficacy and duration of response to alpelisib in three palbociclib-resistant PDX models, irrespective of whether the resistance was intrinsic or acquired. Notably, two of the models were resistant to palbociclib plus fulvestrant. Remarkably, the combination induced robust tumor regression in the HBCx-86 PDX model that was resistant to both alpelisib and onvansertib monotherapies. The HBCx-134palboR31 PDX model, which displayed pronounced tumor regression with the combination, was established from a patient who had been treated with alpelisib. Overall, the effectiveness of onvansertib in enhancing the effect of alpelisib in these therapy-resistant models emphasizes its clinical utility in patients resistant to CDK4/6i, PI3K pathway inhibitors, and endocrine therapy.

In terms of toxicity, onvansertib has been shown to be well tolerated with reversible hematological toxicities, while alpelisib treatment leads to hyperglycemia in patients [15,55,56]. The non-overlapping toxicities of onvansertib and alpelisib provide a supporting rationale for their combination use in the clinical setting. Concurring with this, our *in vivo* studies validated the tolerability of the onvansertib and alpelisib combination.

The synergistic combination effect of onvansertib that we observed was not unique to alpelisib. Onvansertib enhanced the activity of another PI3Kα inhibitor, inavolisib, which is currently being investigated in phase 3 trials. In a recent study, we have identified that the effectiveness of the AKT inhibitor ipatasertib can be enhanced by onvansertib for prostate cancer therapy [33]. It would be interesting to test the efficacy of onvansertib combined with the newly approved AKT inhibitor capivasertib in HR+ breast cancer. Overall, these findings attest to the potential of onvansertib to be combined with inhibitors targeting the PI3K/AKT pathway across indications.

The combined treatment of onvansertib and alpelisib caused marked cell cycle arrest at the G2/M phase in PI3K-activated HR+ breast cancer cell lines. This led to an increase in apoptosis with the combination, both *in vitro* and *in vivo*, as indicated by apoptotic hallmarks such as morphological changes, DNA fragmentation, or PARP protein cleavage. Further studies are needed to determine the factors driving the apoptotic phenotype. The simultaneous inhibition of the PLK1 and PI3K pathways and the sustained suppression of the PI3K/AKT signaling could contribute to the synergistic effect of the combination. However, considering the multiple pathways regulated by PI3K/AKT signaling, further studies are required to have a comprehensive understanding of the underlying molecular mechanisms.

## 5. Conclusions

Our preclinical studies demonstrate the synergistic efficacy of the PI3Kα inhibitor alpelisib and the PLK1 inhibitor onvansertib in *PIK3CA*-mutant HR+ breast cancer cell lines and PDXs that are resistant to ET and palbociclib. Given that approximately 70% of breast cancers are HR+ and eventual resistance to first-line therapies is common, this study is highly relevant. The findings support further investigation of this regimen for *PIK3CA*-mutant HR+ breast cancer patients who show resistance to ET and CDK4/6i or cross-resistance to PI3Kα inhibitors.

## Data Availability

The data presented in this study are available in this article (and Supplementary Material).

## Supplementary Materials

The following supporting information can be downloaded at https://www.mdpi.com/article/10.3390/cancers16193259/s1, Table S1: List of antibodies used in this study; Table S2: *PIK3CA* and *PTEN* mutational status and IC_50_ values of onvansertib and alpelisib in the HR+ cell lines; Figure S1: Onvansertib and alpelisib synergistically inhibits the viability of PI3K-activated HR+ breast cancer cell lines; Figure S2: Combination of inavolisib and onvansertib displays synergistic effect in HR+ breast cancer cell lines; Figure S3: Combination of alpelisib and onvansertib suppresses PI3K-AKT signaling; Figure S4: PLK1 inhibition induced S-phase arrest in EFM-19; Figure S5: Combination of onvansertib and alpelisib is well tolerated *in vivo*; Figure S6: Whole images of Simple Western.
