# Supplementary material for: PLK1 Inhibitor Onvansertib Enhances the Efficacy of Alpelisib in PIK3CA-Mutated HR-Positive Breast Cancer Resistant to Palbociclib and Endocrine Therapy: Preclinical Insights
Source: Cancers (Basel). 2024 Sep 25;16(19):3259. doi: 10.3390/cancers16193259 (PMC11476299; doi:10.3390/cancers16193259)
Supplement: Supplementary file 1 [file cancers-16-03259-s001.zip › cancers-3156913-supplementary.pdf]

Supplementary Methods

PLK1 siRNA transfection.

EFM-19 cells were reverse transfected with 25 nM or 50 nM concentration of siGENOME human SMARTpool control siRNA (Dharmacon, catalog no. D-001810-10-05) or siGENOME human SMARTpool PLK1 siRNA (Dharmacon, catalog no. L-003290-00-0005) using Lipofectamine RNAiMAX (Invitrogen #13778) reagent. After 48 hours, protein lysates are prepared and PLK1 protein expression was analyzed according to the protocol detailed in the materials and methods section. Cell cycle analysis after DAPI staining was performed as detailed in the materials and methods section.

Table S1: List of antibodies used in this study.

Antibody	**Supplier**	**Catalog number**	**Dilution**
AKT	Cell Signaling	9272	1:50
β-ACTIN	Santa Cruz Bio	sc-47778	1:100
Cleaved-PARP	Cell Signaling	5625	1:50
GSK3β	Cell Signaling	9315	1:50
PARP	Cell Signaling	9532	1:50
Phospho-AKT (Ser473)	Cell Signaling	4060	1:50
Phospho-GSK3β (Ser9)	Cell Signaling	9336	1:50
Phospho-S6 ribosomal protein (Ser240/244)	Cell Signaling	2215	1:50
Phospho-TCTP (Ser46)	Cell Signaling	5251	1:50
PLK1	Cell Signaling	4513	1:50
S6 ribosomal protein (S6)	Cell Signaling	2217	1:50
TCTP	Cell Signaling	5128	1:50
Anti-rabbit secondary	Bio-Techne	042-206	1:1

Table S2: *PIK3CA* and *PTEN* mutational status and IC_{50} values of onvansertib and alpelisib in the HR+ cell lines.

**Cell line**	**Mutational Status**		**IC_{50} (nM)**	
	PIK3CA	***PTEN***	**Onvansertib**	**Alpelisib**
**MCF7**	Mut (E545K)	WT	68 ± 5	273 ± 27
**T-47D**	Mut (H1047R)	WT	211 ± 4	216 ± 3
**EFM-19**	Mut (H1047L)	WT	23 ± 2	185 ± 14
**CAMA-1**	WT	Loss	58 ± 6	> 1000
**ZR-75-1**	WT	Loss	135 ± 18	> 1000
**MCF7/164R-7 (Fulvestrant-resistant)**	Mut (E545K)	WT	64 ± 1	288 ± 20

Note: Mut=mutant; WT=wild-type

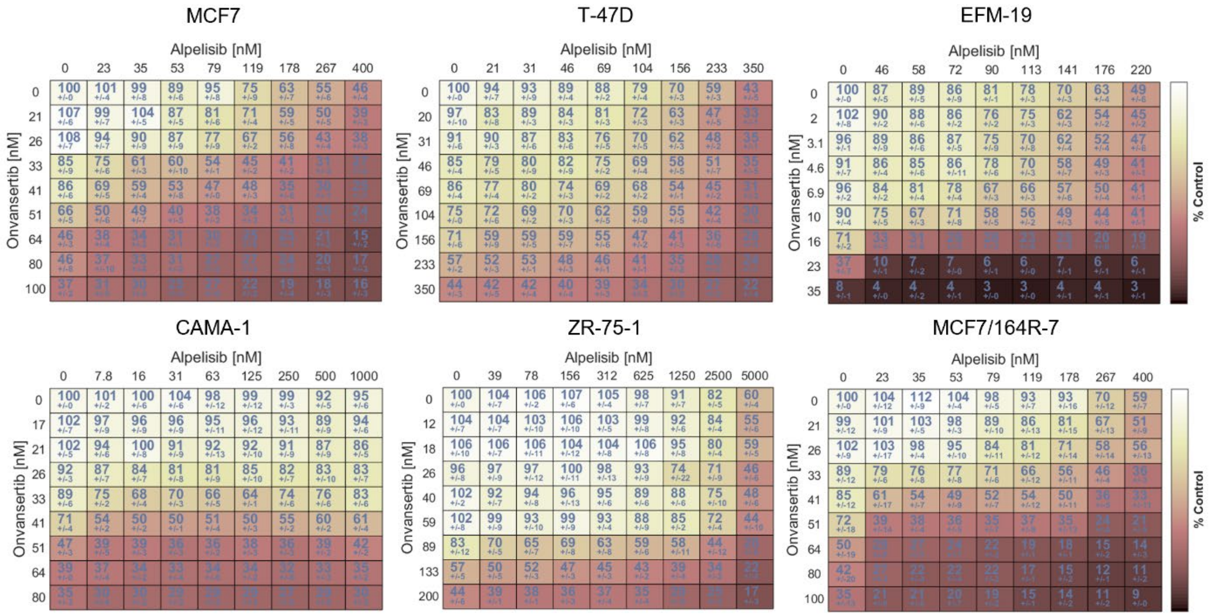

Figure S1: Onvansertib and alpelisib synergistically inhibits the viability of PI3K-activated HR+ breast cancer cell lines. Cell viability was assessed after 6-7 days of treatment with onvansertib (Onv), alpelisib (Alp) or the combination of Onv + Alp at the indicated concentrations. Percent inhibition dose response matrices of the onvansertib and alpelisib single agents and the combinations are shown (n=3).

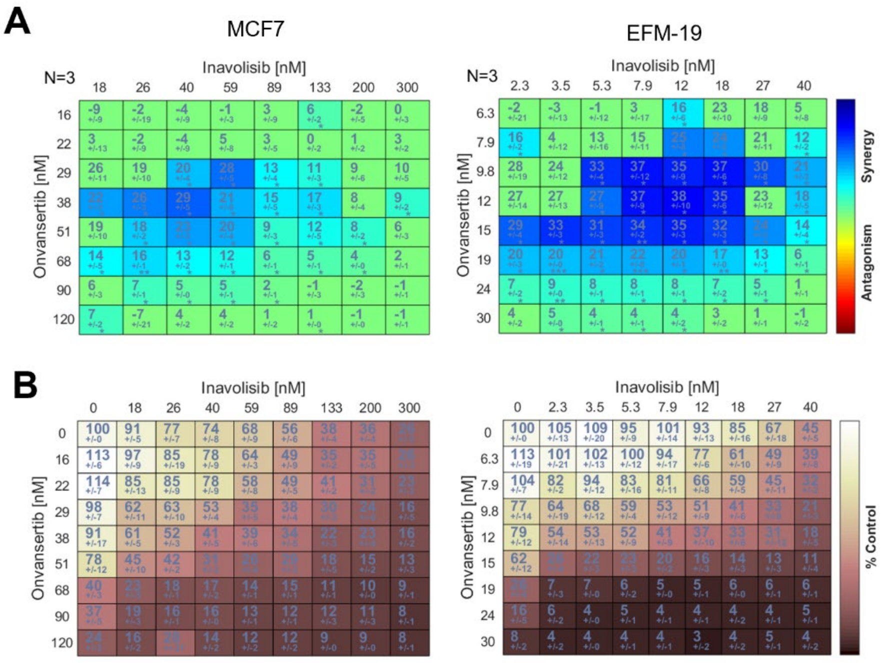

Figure S2: Combination of inavolisib and onvansertib displays synergistic effect in HR+ breast cancer cell lines. **(A)** The cells were seeded in 384-well plates and exposed to 8 different concentrations of inavolisib and onvansertib, as well as the possible combinations of the two drugs. Cell viability was assessed after 6-7 days, and resultant data was then subjected to synergy analysis using Combenefit software. Heatmaps of the combination responses for onvansertib and inavolisib based on Bliss synergy model analysis are shown. Blue color indicates synergistic interaction. **(B)** Percent cell viability inhibition dose response matrices of the onvansertib and inavolisib single agents and the combinations are shown (n=3).

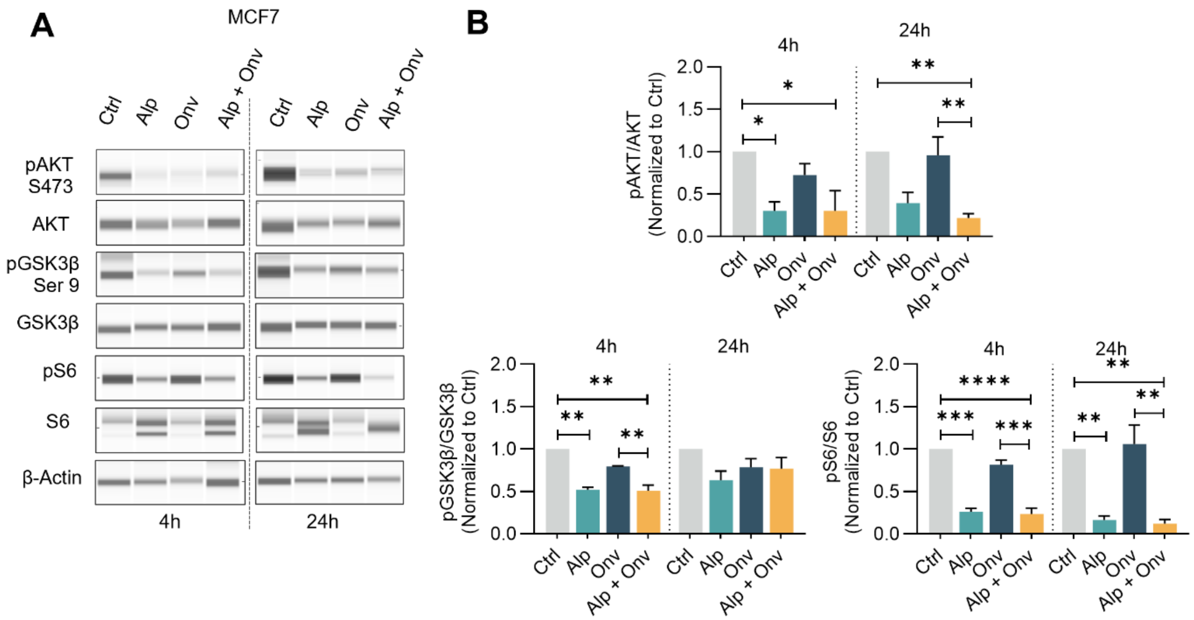

Figure S3: Combination of alpelisib and onvansertib suppresses PI3K-AKT signaling **(A)** MCF7 cells were treated with DMSO vehicle (Ctrl), Onv (25 nM), Alp (200 nM) or Onv + Alp for 4h or 24h and pAKT-Ser473, AKT, GSK3β, pGSK3β-Ser9, Total S6, pS6-Ser240/244 (pS6), and β-Actin protein expression was analyzed by Protein Simple Western. **(B)** Densitometric ratio of the phosphorylated proteins to total protein expression-levels normalized to DMSO control are plotted from three experiments as mean ± SEM. One-way ANOVA was used to compare the means. Asterisks indicate significance (*$p<0.05$, **$p<0.01$, ***$p<0.001$, ****$p<0.0001$).

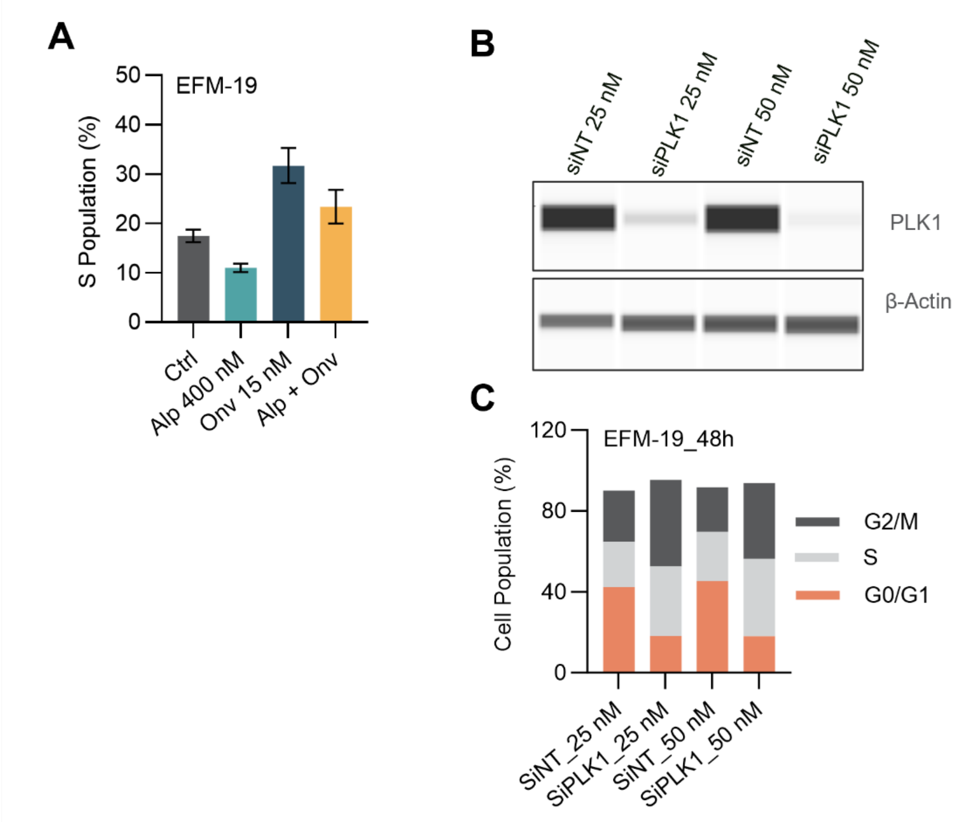

Figure S4: PLK1 inhibition induces S-phase arrest in EFM-19. **(A)** Effect of alpelisib (Alp) and onvansertib (Onv) and the combination (Alp + Onv) on S-phase distribution of EFM-19. The percentage of cells in S phase is plotted as mean ± SEM from 3 independent experiments. **(B)** PLK1 expression was analyzed by Protein Simple Western in cells transfected with 25 nM and 50 nM of non-target siRNA (siNT) or siPLK1. β-Actin was used as a loading control. **(C)** Cell cycle phase distribution of cells transfected with non-target siRNA (siNT) or siPLK1. The percentage of cells in the G1, S and G2/M phases are plotted.

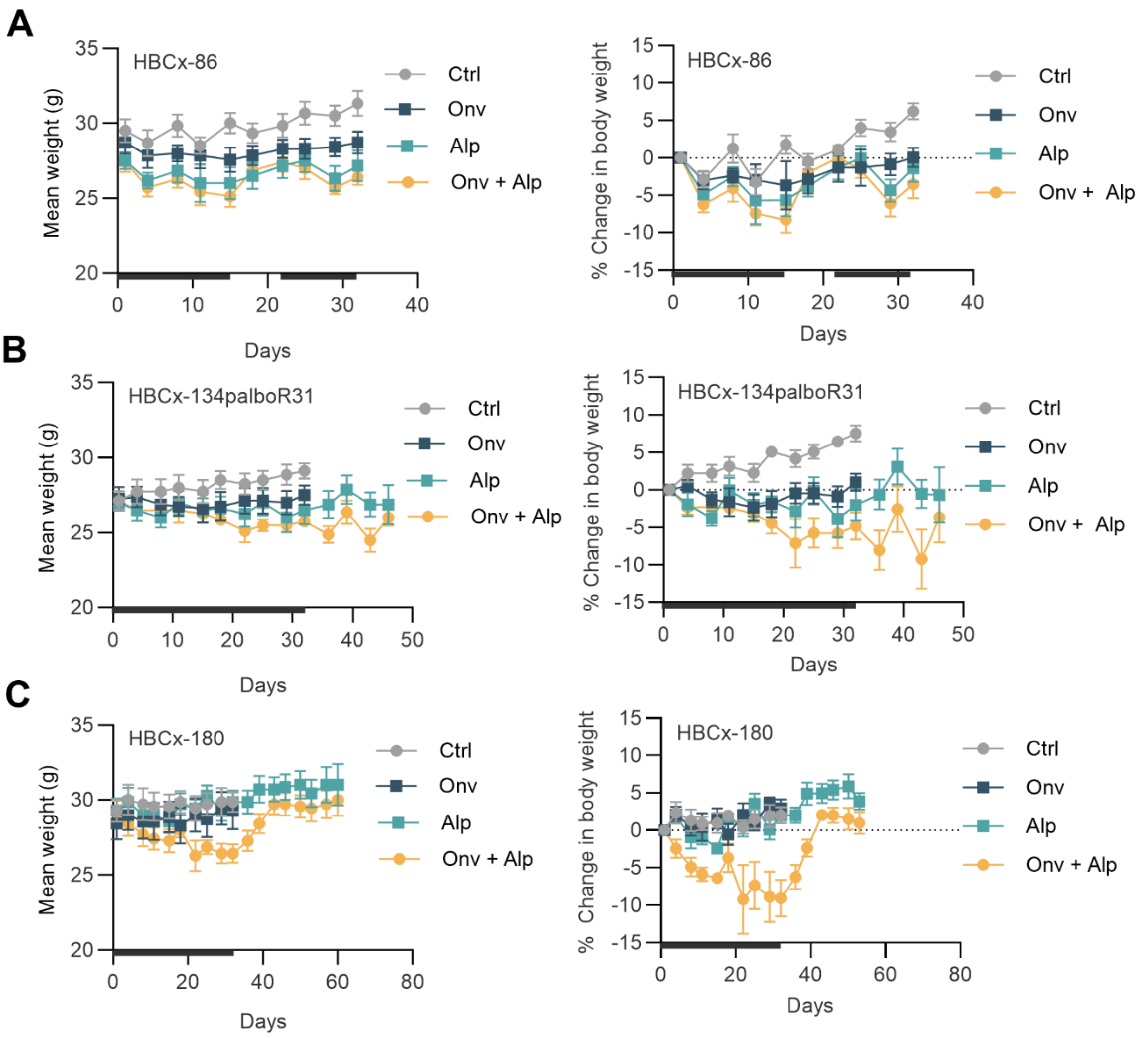

Figure S5: Combination of onvansertib and alpelisib is well tolerated *in vivo*. (**A-C**) Mean body weight and percentage change in body weight mice treated with vehicle (Ctrl), onvansertib (Onv), alpelisib (Alp) or combination of Alp and Onv for the indicated duration (**—**). **(A)** HBCx-86, **(B)** HBCx-134palboR31 and **(C)** HBCx-180 PDX models.

Figure S6: Whole images of Simple Westerns.

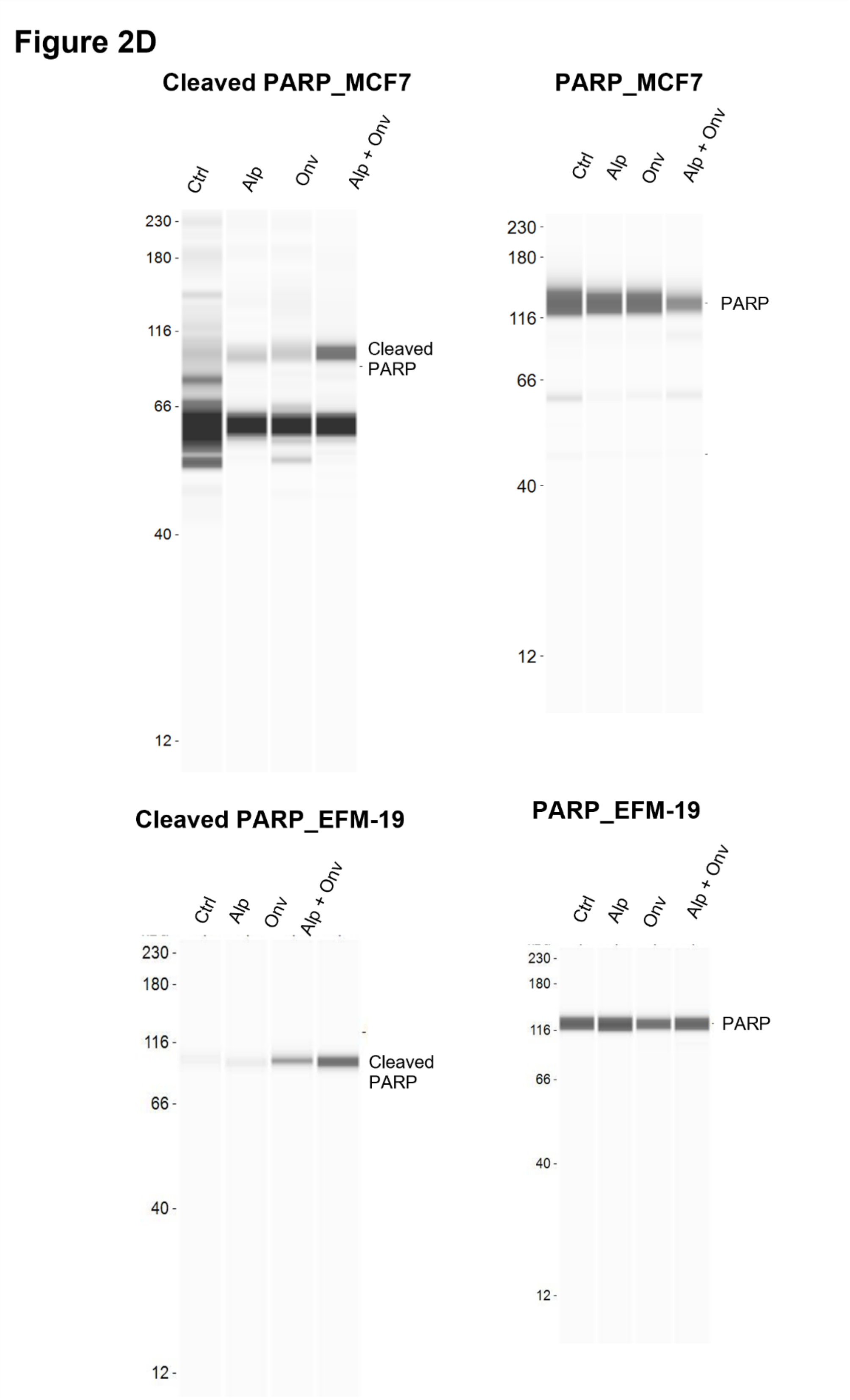

Figure 4A

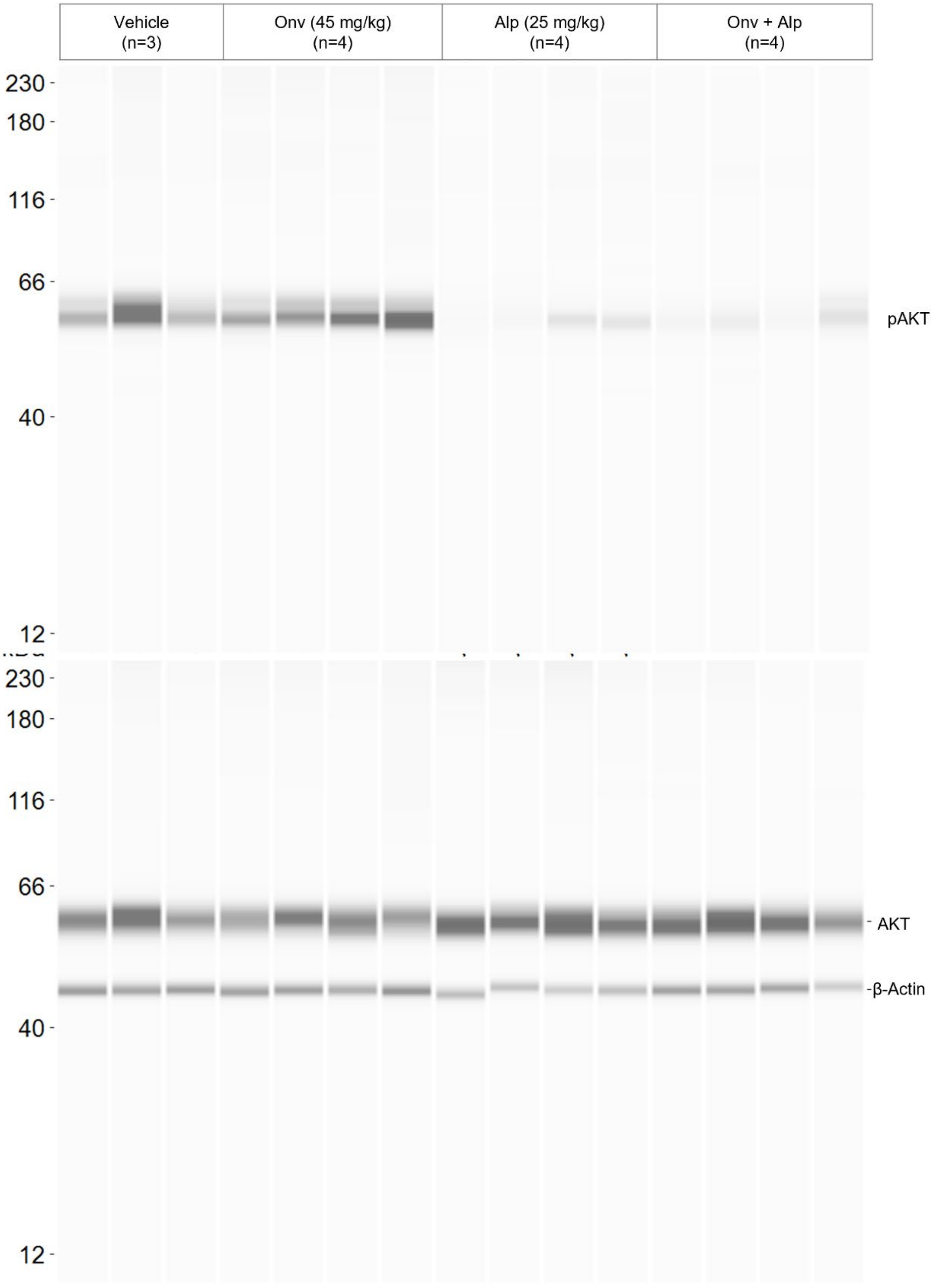
Vehicle (n=3)
Onv (45 mg/kg) (n=4)
Alp (25 mg/kg) (n=4)
Onv + Alp (n=4)
230
180
116
66
40
12
pAKT
230
180
116
66
40
12
AKT
β-Actin

Figure 4A

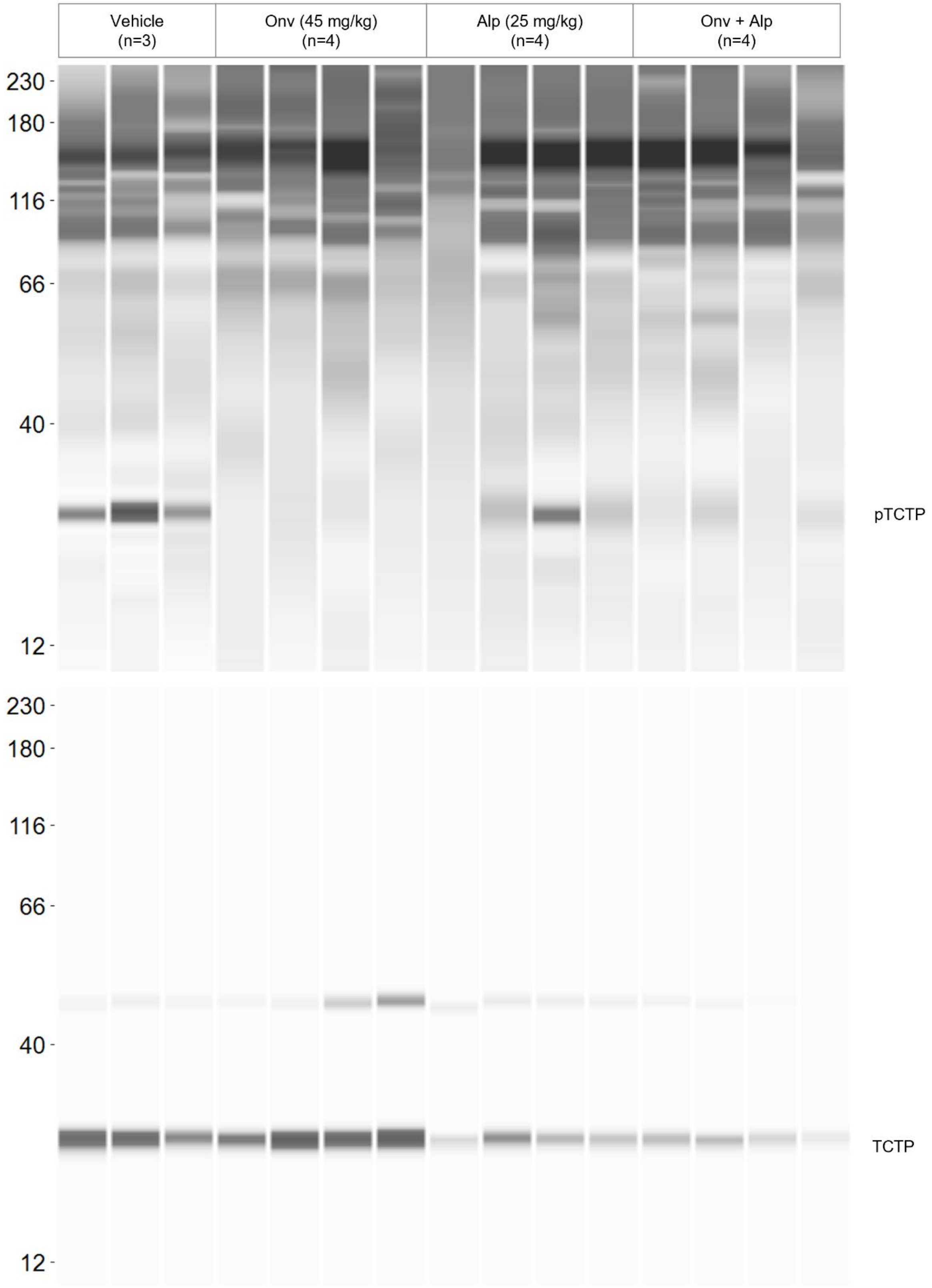
Vehicle (n=3)
Onv (45 mg/kg) (n=4)
Alp (25 mg/kg) (n=4)
Onv + Alp (n=4)
230
180
116
66
40
12
pTCTP
230
180
116
66
40
12
TCTP

Figure 4F

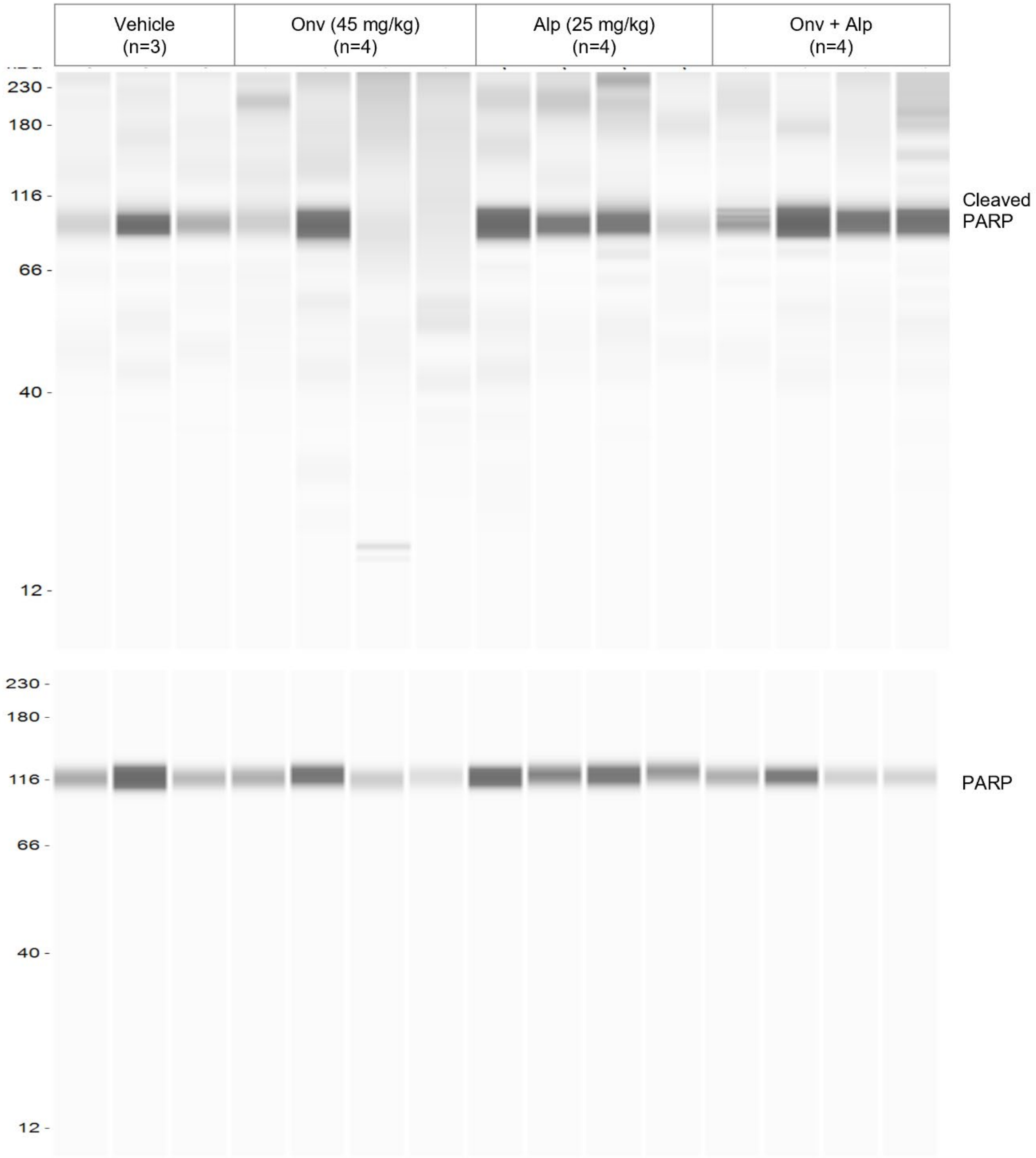

Vehicle (n=3)
Onv (45 mg/kg) (n=4)
Alp (25 mg/kg) (n=4)
Onv + Alp (n=4)
230
180
116
66
40
12
Cleaved PARP
230
180
116
66
40
12
PARP

Figure S3A

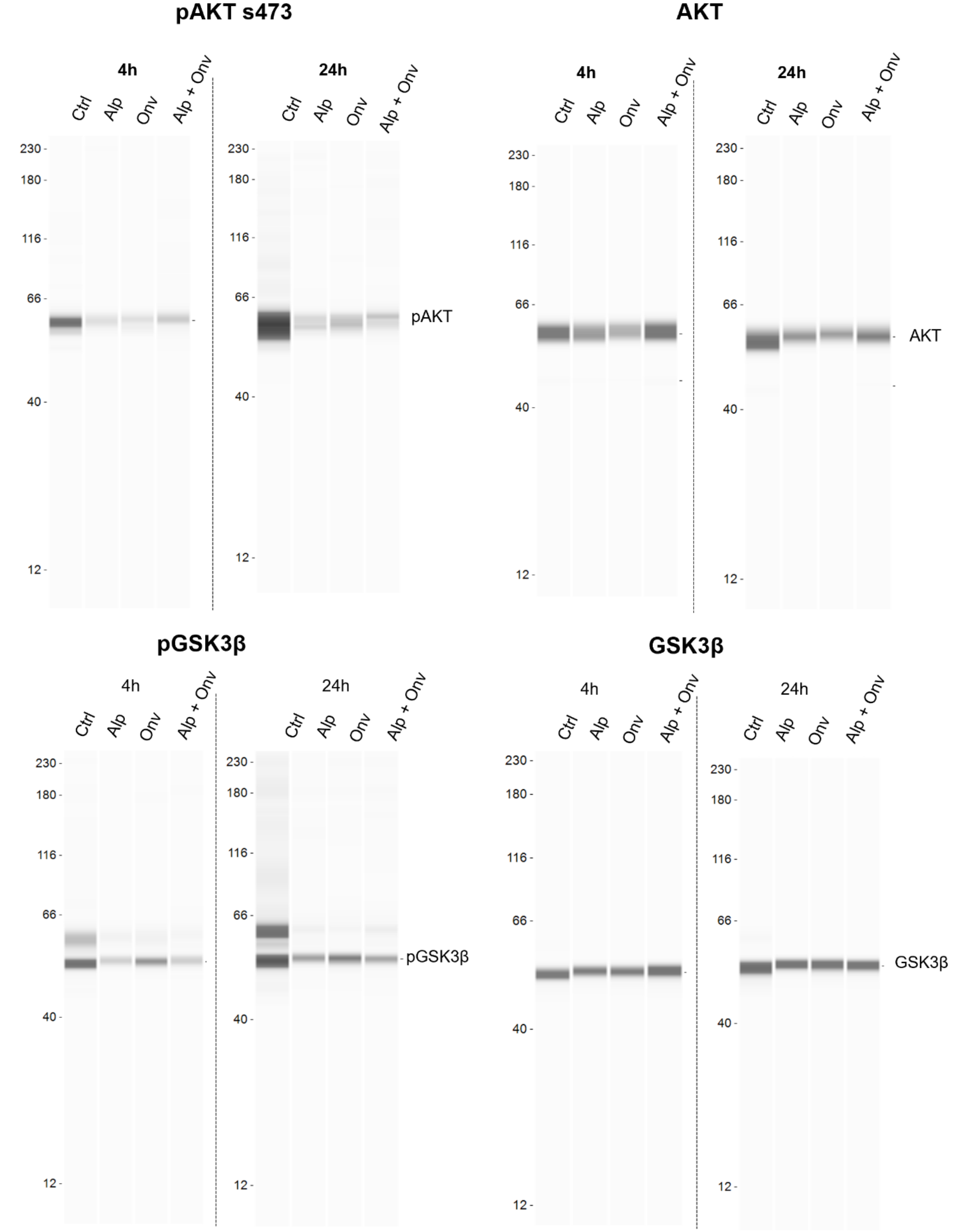
pAKT s473
AKT
pGSK3β
GSK3β
4h
24h
Ctrl
Alp
Onv
Alp + Onv
230
180
116
66
40
12
pAKT
AKT
pGSK3β
GSK3β

Figure S3A

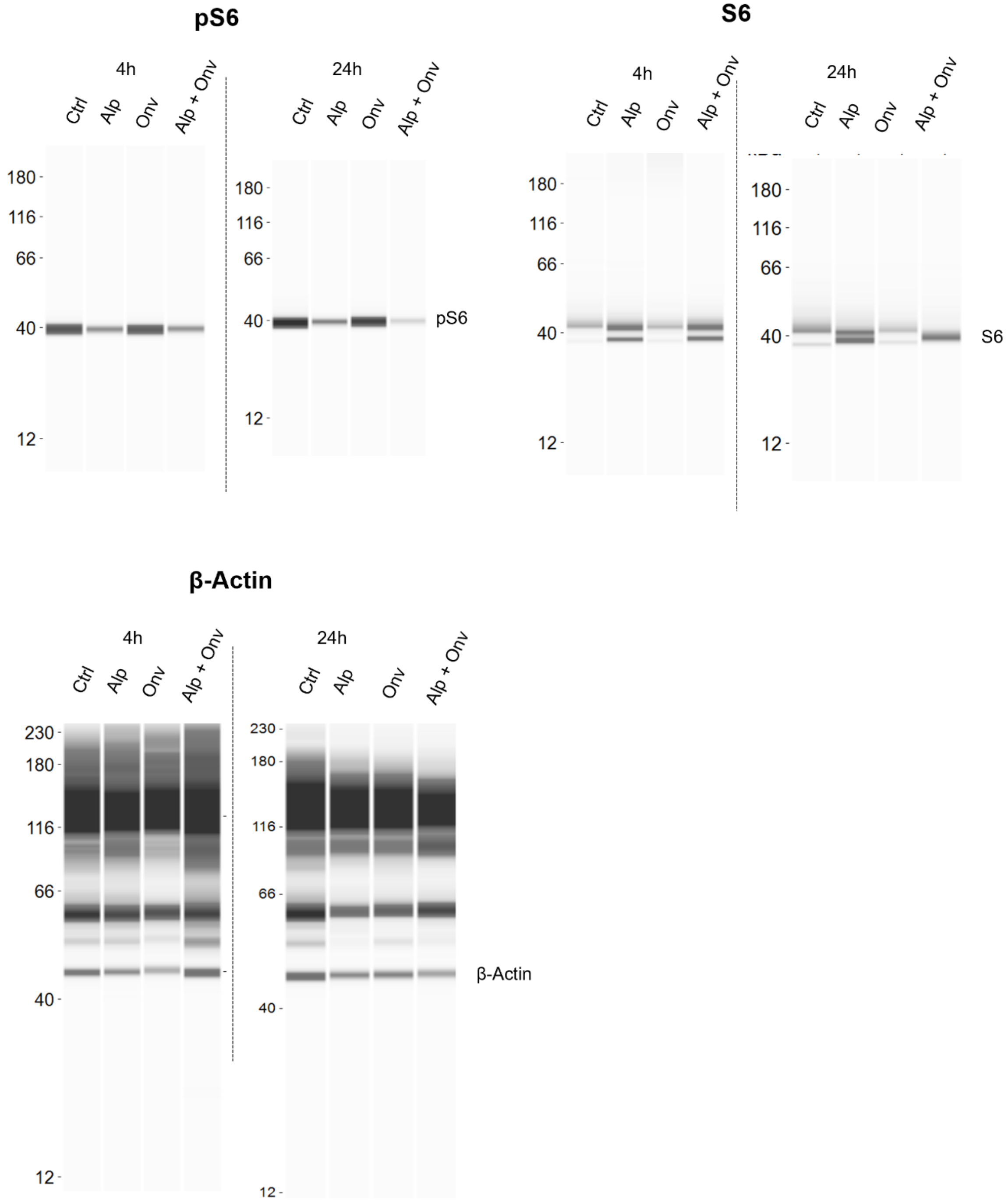

pS6
4h
24h
Ctrl
Alp
Onv
Alp + Onv
180
116
66
40
12
pS6
S6
S6
β-Actin
230
β-Actin

Figure S4B

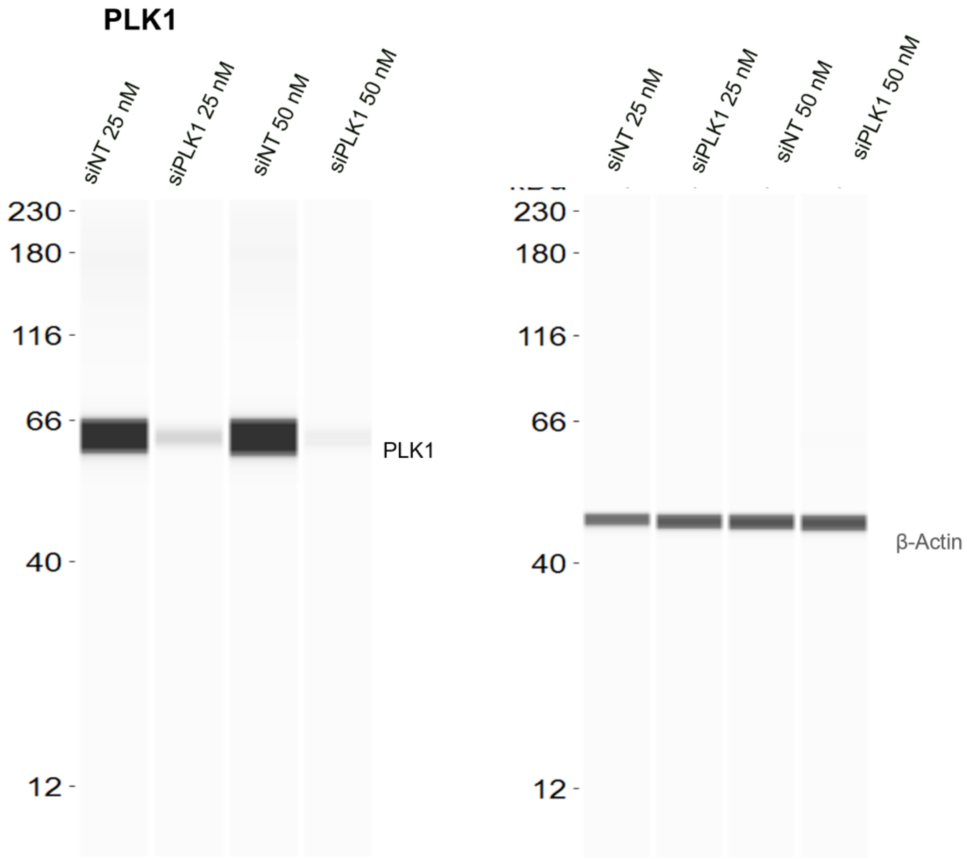
PLK1
siNT 25 nM
siPLK1 25 nM
siNT 50 nM
siPLK1 50 nM
230
180
116
66
40
12
PLK1
siNT 25 nM
siPLK1 25 nM
siNT 50 nM
siPLK1 50 nM
230
180
116
66
40
12
β-Actin
